# Prevalence of idiopathic normal pressure hydrocephalus: A prospective, population-based study

**DOI:** 10.1371/journal.pone.0217705

**Published:** 2019-05-29

**Authors:** Johanna Andersson, Michelle Rosell, Karin Kockum, Otto Lilja-Lund, Lars Söderström, Katarina Laurell

**Affiliations:** 1 Department of Pharmacology and Clinical Neuroscience, Neurology, Umeå University, Umeå, Sweden; 2 Unit of Research, Development and Education, Östersund, Sweden; Goethe University Hospital Frankfurt, GERMANY

## Abstract

**Background:**

Idiopathic normal pressure hydrocephalus (iNPH) causing gait impairment, dementia and urinary incontinence among the elderly, is probably under-diagnosed and under-treated. Despite being known since the 1960s, there is still a lack of prospective, population-based studies on the prevalence of iNPH. Such studies are warranted to minimize selection bias and estimate the true prevalence of the disease.

**Methods:**

The prevalence of iNPH was determined in a randomly selected sample of residents, aged 65 years and older, in the Swedish county of Jämtland. Out of 1,000 individuals invited to participate, 673 (67.3%) completed a questionnaire with seven questions on iNPH symptoms. A subgroup, with and without self-reported symptoms, participated in clinical and radiological evaluations and were diagnosed according to international guidelines. Measurement of cerebrospinal fluid opening pressure was not performed as it was considered too invasive.

**Results:**

Those who reported at least two symptoms in the questionnaire (n = 117) and 51 randomly selected individuals with 0–1 symptom participated in further examinations. Out of them, 25 individuals received the diagnosis probable iNPH according to American-European guidelines (except for the criterion of CSF opening pressure) corresponding to a prevalence of 3.7%. The prevalence of iNPH was four times higher among those aged 80 years and older (8.9%) than among those aged 65–79 years (2.1%) (p <0.001). The difference in prevalence between men (4.6%) and women (2.9%) was not significant (p = 0.24). When iNPH was diagnosed according to the Japanese guidelines the prevalence was 1.5%

**Conclusions:**

In this prospective, population-based study the prevalence of iNPH was 3.7% among individuals 65 years and older, and more common in the higher age group, 80 years and above. INPH should be increasingly recognized since it is a fairly common condition and an important cause of gait impairment and dementia among the elderly that can be effectively treated by shunt surgery.

## Introduction

Globally, a growing number of elderly people live within health care facilities due to disabilities such as dementia and/or gait disorders [1]. In a study from the United States, about 14% of elderly people in nursing homes suffer from the progressive neurological disorder idiopathic normal pressure hydrocephalus (iNPH) with the cardinal symptoms of gait impairment, cognitive decline and urinary incontinence [2]. The disorder is sometimes mistaken for Alzheimer’s or Parkinson’s disease but has characteristic imaging findings, such as ventriculomegaly in combination with narrowing of the callosal angle [3, 4]. The condition can be effectively treated with surgery, in fact, studies have found that up to 80% of patients improve with insertion of a ventriculoperitoneal shunt [5]. However, the outcome of shunt surgery worsens with the progression of the disease, highlighting the importance of early diagnosis [6].

Even though the disorder has been known since the 1960s [7] the prevalence in the general population is still unclear. The publications of two different international diagnostic guidelines in 2004 and 2005 [8, 9] were followed by an increasing number of studies, with prevalence figures ranging from 0.5% to 2.9% among those aged 65 and older [10–16]. A few studies have reported on prevalence numbers in relation to age and sex, suggesting iNPH to be more prevalent in the older age category [11, 13, 15], with only one study showing a predominance among men [13]. In addition to varying definitions of iNPH, previous studies have limitations such as the use of clinical-based materials or retrospective data from cohorts followed for other diseases such as dementia [17].

Despite the opportunity to improve the symptoms by shunt surgery, previous prevalence studies indicate that iNPH is under-diagnosed and under-treated. Prospective, population-based studies are warranted to minimize selection bias and estimate the true prevalence of the disease, as well as age and sex differences. Due to the lack of such studies we aimed to estimate the overall prevalence of iNPH, as well as age and sex differences, among individuals aged 65 years or older from the general population.

## Materials and methods

### Study population

**Fig 1:**
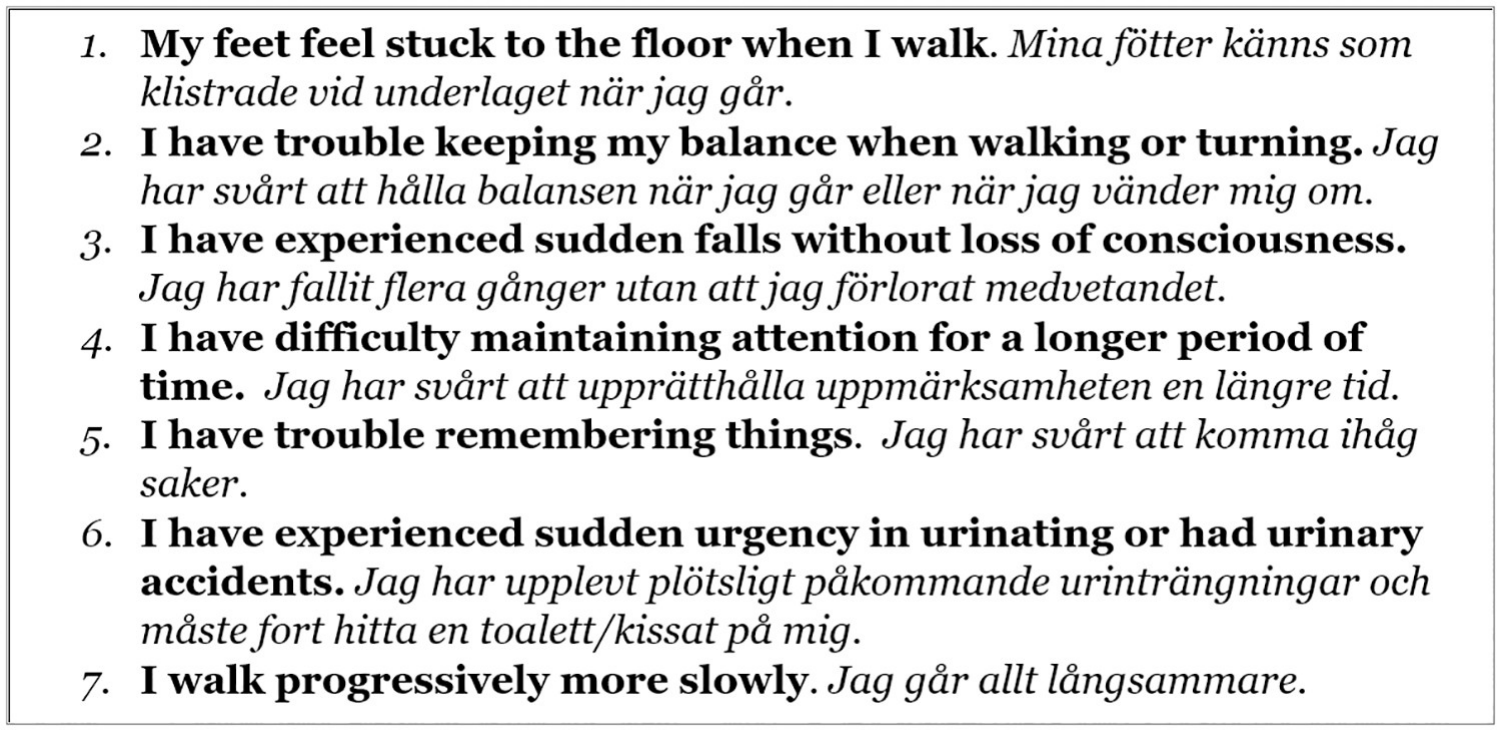
Screening questionnaire (“yes” or “no” answers) in English (bold) and Swedish (italics).

**Fig 2:**
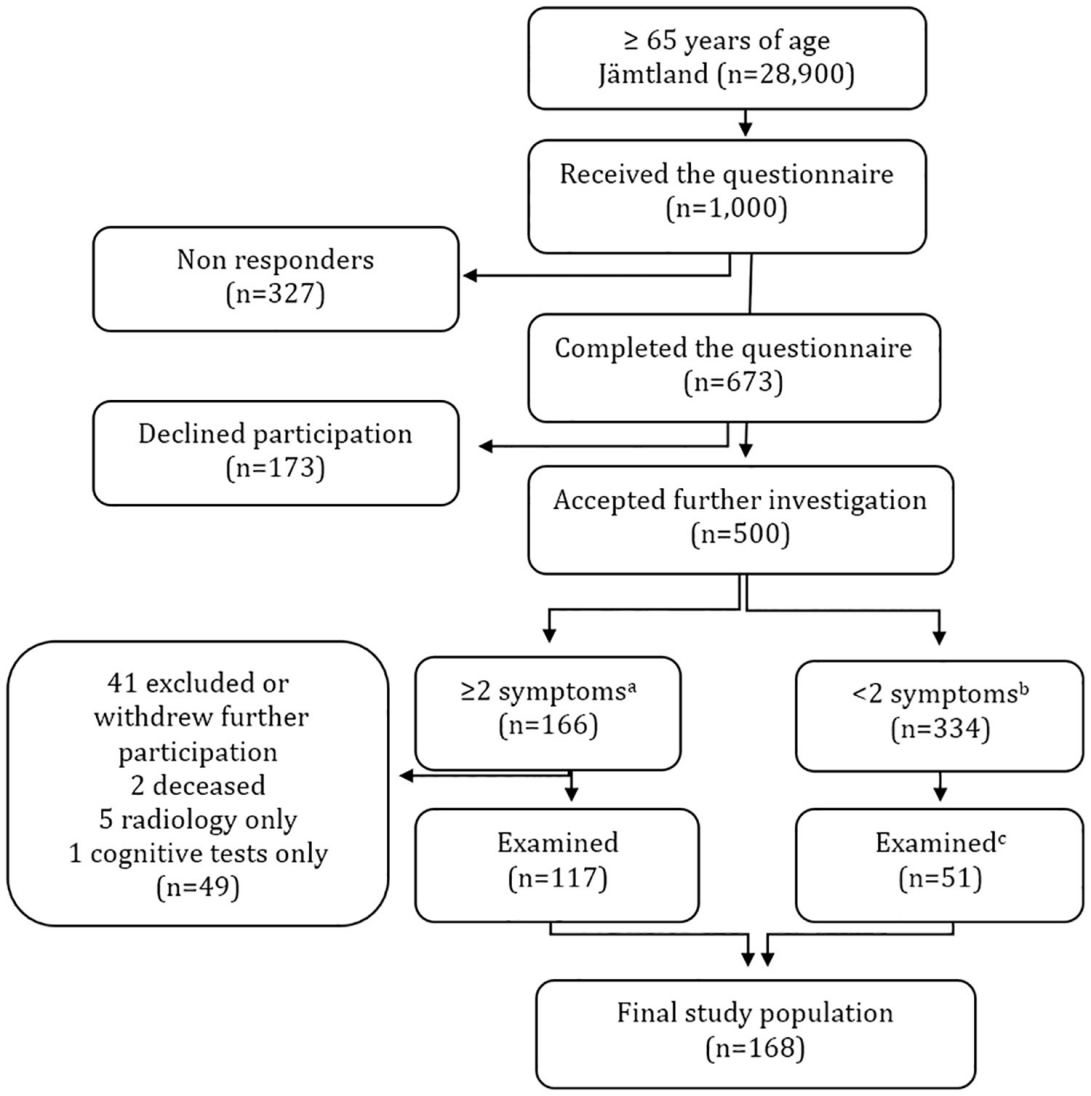
Flow chart of sample selection. Note: ^a^ Gait disturbance mandatory. ^b^ Including 49 individuals reporting two symptoms of incontinence and cognition but no gait disturbance. ^c^ Randomly selected, including five individuals reporting two symptoms on incontinence and cognition but no gait disturbance.

During the autumn of 2014 a prevalence study on iNPH was conducted in the county of Jämtland, Sweden. In 2014, Jämtland had a total population of 127,000 of which, 28,900 was 65 years and older. In this age group there was a predominance of women (52.9%) and even more so (61.0%) among those aged 80 years and older [18]. Using the Swedish population register [19], 1,000 individuals from Jämtland, aged 65 years and older, were randomly selected and invited to participate in the study. Before starting, the study was mentioned in the local radio and the newspaper. The selected sample of residents received a simple questionnaire based on an American precursor [20], assessing typical symptoms of iNPH; four regarding gait and postural stability, one on continence and two on cognition, see Fig 1. Relatives were encouraged to assist in filling out the questionnaire when needed. A reminder was sent about two weeks later. All individuals who reported gait or balance impairment together with one of the other symptoms were invited for further evaluation. Exclusion criteria were severe medical conditions sufficient to explain the symptoms, e.g. a known brain tumour or a traumatic brain injury. A randomly selected group with less than two symptoms was included for comparison, see Fig 2 for more details on inclusion and dropouts.

The study was approved by the Regional Ethical review board at Umeå University in 2014 (Dnr 2014/180-31) and the participants gave written informed consent.

### Clinical and radiological examination

All study participants underwent clinical and neuropsychological examinations as well as a computerized tomography (CT) of the brain within six months of the examinations. Two radiologists independently assessed the CT-scans and the radiological and clinical evaluations were made blinded to one another. Radiological parameters with typical features of iNPH were measured, for example ventriculomegaly (Evans’ index) and the callosal angle [21].

The clinical evaluation included medical history and a neurological examination. Gait and balance were tested with Time Up and Go (TUG), and 10 m walking test as well as ordinal rating-scales used in previous iNPH research [22, 23]. The neuropsychological examination included Rey Auditory Verbal Learning Test (RAVLT), and Mini-Mental State Examination (MMSE) [23, 24]. Participants rated urinary symptoms such as urgency and incontinence on an ordinal scale [23]. The degree of disability was rated using modified Ranking Scale (mRS)[25].

The American-European guidelines as well as the Japanese guidelines (2^nd^ edition) were used to classify the participants [9, 26] see Table 1. However, we excluded the CSF opening pressure criterion for probable iNPH as a lumbar puncture was considered too invasive. Thus, we will here use the term modified probable iNPH (*m*-probable iNPH) for the most likely category of iNPH according to the American-European guidelines.

**Table 1:** Diagnostic criteria according to the two international guidelines.

**A. American-European guidelines [9]**			
	**Probable**^a^	**Possible**	**Unlikely**
**Clinical features**:	Gait/balance disturbance and at least one of the following:	Symptoms of either:	No component in the clinical triad or symptoms explained by other causes
	a) Cognitive impairment	a) Incontinence and/or cognitive impairment in the absence of gait/balance disturbance	
	b) Urinary incontinence/urgency	b) Gait disturbance alone	
**Brain imaging**	Ventriculomegaly (EI >0.3) and at least one of the following:	Ventriculomegaly (EI >0.3)	No evidence of ventriculomegaly
	a) Narrow callosal angle		
	b) Enlargement of the temporal horns		
	c) Periventricular signal changes not attributable to ischemic changes or demyelination		
**B. Japanese guidelines (2nd edition) [26]**			
	**Possible with MRI support**	**Possible**	**Unlikely**
**Clinical features**	At least two of the clinical triad: Gait disturbance, cognitive impairment and urinary incontinence	At least two of the clinical triad: Gait disturbance, cognitive impairment and urinary incontinence	None of this
**Brain imaging**	Ventriculomegaly (EI >0.3) and the following:	Ventriculomegaly (EI >0.3)	No evidence of ventriculomegaly
	a) Narrowing of the sulci over the high convexity/DESH [4]		

^a^ Not including the criteria CSF opening pressure < 25 cm H_2_0

EI = Evans Index, DESH = Disproportionately enlarged subarachnoid space hydrocephalus.

To enable large epidemiological studies, the Japanese guidelines include the diagnosis “possible iNPH with MRI support” which relies more upon radiological findings and do not require measurements on CSF opening pressure. These guidelines also contain a diagnosis called “definite iNPH” for those who respond to shunting.

### Statistical analyses

Descriptive statistics were used for the epidemiological data. The prevalence was calculated by dividing the number of individuals diagnosed with iNPH by the total number of people who returned completed questionnaires.

Differences regarding age and sex between questionnaire responders and non-responders, participants and non-participants in clinical examinations were analysed with the Mann-Whitney U test and the chi-square test, respectively.

Differences in the prevalence of iNPH in relation to age and sex were tested with the chi-square test, and Fischer’s exact test when appropriate.

Statistical analyses were performed using SPSS (version 24). The level of significance was set to <0.05.

## Results

### Study population

Out of the 1,000 invited individuals, 673 returned completed questionnaires corresponding to a response rate of 67.3%, see Fig 2. The median age of the responders was 72.0 years (IQR 11) with the majority (76.5%) under the age of 80. No significant differences were found in sex distribution; 48.4% were men (median 72.0 years, IQR 11) and 51.6% women (median 72.5 years, IQR 10).

The non-responders (n = 327) were older (median 75.0, IQR 12, p<0.001) and significantly more often women (58.4%, p<0.05) than the responders. Those who declined further participation (n = 173) were also significantly older (median 75.5, IQR 14) than those who accepted (n = 500) (median 72.0, IQR 10, p<0.001) but there was no significant difference in sex.

Age and sex did not differ significantly between those with two or more symptoms who participated in further examinations (n = 117) and drop-outs (n = 49). Similarly, those with less than two symptoms that were randomized for further examinations (n = 51) did not differ in age and sex from those who were not examined (n = 283).

In total 168 individuals, 117 with symptoms and 51 randomly selected controls were taken in for further examination at the neurology and radiology department of Östersund’s hospital from August 2014 to October 2015. The characteristics of the investigated sample are presented in Tables 2 and 3.

**Table 2:** The age and sex distribution of the study sample (n = 168), broken down into diagnosis (American-European guidelines).

	Unlikely iNPH (n = 119) n (%)	Possible iNPH (n = 24) n (%)	*m-*Probable iNPH (n = 25) n (%)
< 80 yrs	102 (85.7)	19 (79.2)	11 (44.0)
≥80 yrs	17 (14.3)	5 (20.8)	14 (56.0)
Males	45 (37.8)	16 (66.7)	15 (60.0)
Females	74 (62.2)	8 (33.3)	10 (40.0)

**Table 3:** Characteristics of the study sample (n = 168), broken down into diagnosis (American-European guidelines).

	Unlikely iNPH (n = 119) median (IQR)	Possible iNPH (n = 24) median (IQR)	*m-*Probable iNPH (n = 25) median (IQR)
Age	72.0 (7.0)	73.5 (11.0)	82.0 (11.0)
MMSE	28.0 (2.0)	27.0 (7.0)	27.0 (4.0)
RAVLT (total word count)	33.0 (11.0)	25.0 (11.0)	24.0. (13.0)
mRS	2.0 (2.0)	2.0 (1.0)	3.0 (2.0)
TUG, time (s) / steps	9.9 (3.9)/14.0 (4.5)	11.6 (3.1)/14.5 (4.0)	14.2 (5.3)/18.0 (4.3)
10 m, time (s)/ steps	9.0 (2.4)/16.0 (4.0)	9.4 (2.5)/16.0 (4.0)	11.5 (3.5)/18.5 (4.1)
Continence	2.0 (2.0)	2.0 (2.0)	3.0 (2.0)
Evans’ index	0.27 (0.03)	0.32 (0.01)	0.34 (0.04)

### Prevalence

A total of 25 individuals fulfilled the American-European guidelines for *m-*probable iNPH, 24 for possible iNPH and the remaining 119 were classified as unlikely iNPH, see Table 2. Out of the population of 673 individuals who responded to the questionnaire, this corresponds to a prevalence of *m-*probable iNPH of 3.7%. According to the Japanese guidelines the number with possible iNPH with MRI support or definite iNPH was 10 corresponding to a prevalence of 1.5%. Among those who reported less than two symptoms in the questionnaire, one fulfilled the criteria for *m*-probable iNPH according to American-European guidelines and none for possible iNPH with MRI support or definite iNPH according to the Japanese guidelines.

One of the participants in the study was already treated with a VP-shunt for iNPH, and one received a shunt during the study period with improvement of symptoms. Three of the participants were diagnosed with asymptomatic iNPH having radiological signs but no clinical symptoms, these individuals were included in the group of unlikely iNPH.

### Age and sex differences

The prevalence of *m-*probable iNPH was significantly higher among those aged over 80 years (8.9%) than under (2.1%), (p<0.001), whereas the difference between men (4.6%) and women (2.9%) was non-significant (p = 0.24).

**Fig 3:**
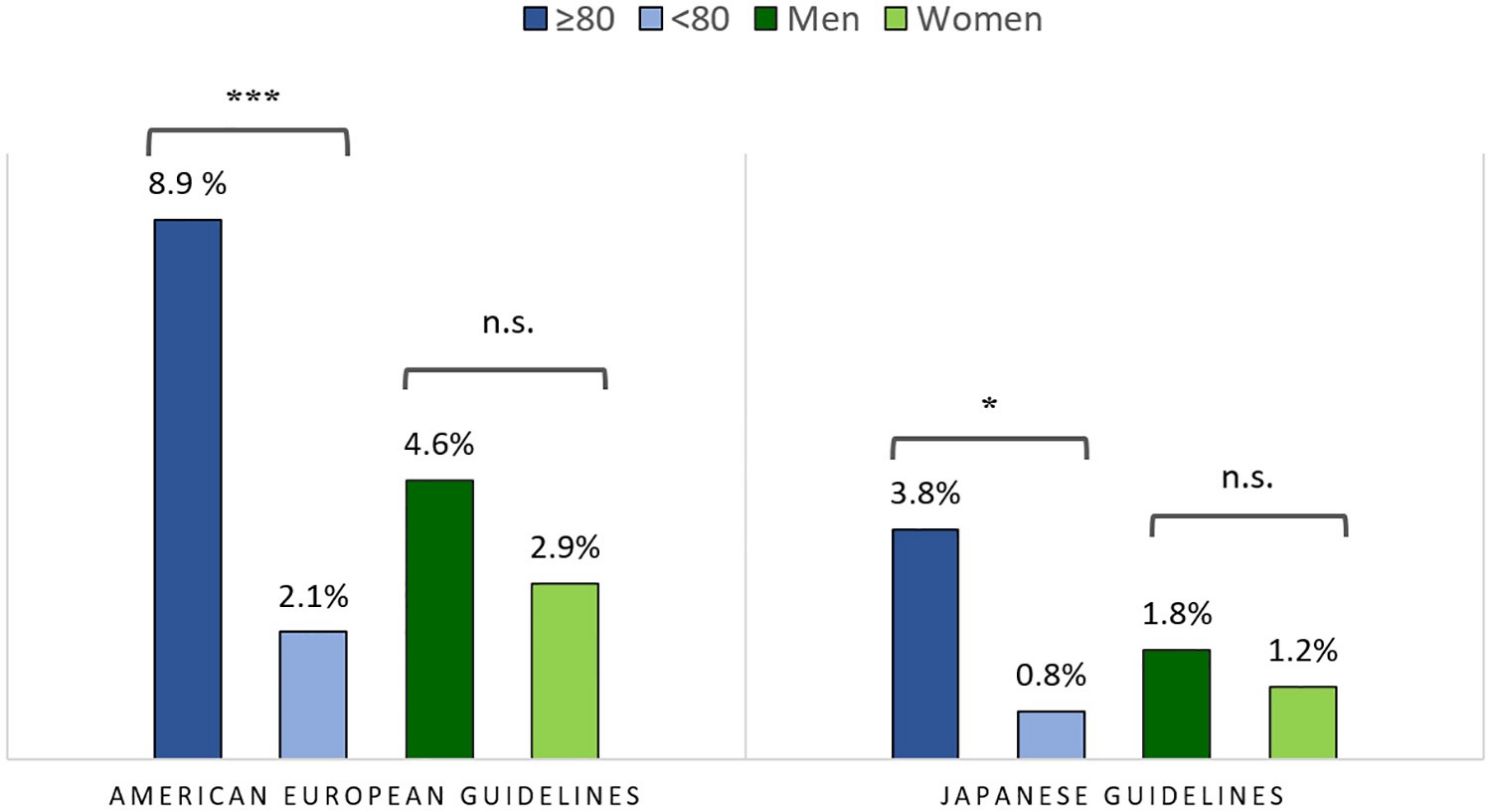
Prevalence of iNPH (possible iNPH excluded) in relation to age and sex with the American-European and Japanese guidelines, respectively.

According to the Japanese guidelines the prevalence of possible iNPH with MRI support and definite iNPH was 3.8% for those over 80 years and 0.8% for those under 80 years (p<0.05) The corresponding prevalence figures for men and women were 1.8% and 1.2% respectively (p = 0.53). The age and sex differences in prevalence of iNPH are presented in Fig 3.

## Discussion

In this prospective, population-based study the estimated prevalence of iNPH among individuals 65 years and older was 3.7% according to a modified version of the American-European guidelines and 1.5% according to the Japanese guidelines. This prevalence figure is higher than in most previous studies, probably because of the prospective screening for the disorder in the general population.

### Age and sex differences

INPH was four times as common among those aged 80 years and older (8.9%) than among those younger than 80 years (2.1%). In accordance, a previous Swedish study reported a pronounced increase in the prevalence of iNPH from 0.2% in those aged 70–79 years to 5.9% among those over the age of 80 [11]. However, the prevalence figures were lower than in the present study, which might be because they used retrospective data from different cohort studies on aging and health.

In the neighbouring country Norway, a much lower prevalence of iNPH, 0.02%, was reported among subjects over 65 years, with a higher prevalence (0.18%) in the older age group of 70–79 years [15]. The low prevalence could probably be due to that participants were collected through advertisements in media and information campaigns directed to primary care physicians. Most likely, the whole population was not reached by these methods, which might have led to a recruitment-bias. The American-European guidelines were used including measurements of lumbar opening pressure in as many as 63 participants.

In Japan, three studies have been conducted on the prevalence of iNPH with quite varying results in spite of similar methodology [10, 12, 16]. Iseki et al. [10] reported a prevalence of 0.51% in 790 individuals, aged 61 or 70–72 years, who participated in a previous study of cerebrovascular risk factors. At a follow-up ten years later, the prevalence was 1.4% among those aged 80 years [14]. Both Hiraoka et al. [16] and Tanaka et al. [12] used data from a previous study on dementia and reported a prevalence of iNPH among those aged 65 and older of 2.9% (n = 170) and 1.4% (n = 567), respectively. To be considered as iNPH in these studies from Japan, MR-imaging signs as well as at least one symptom of iNPH were required.

Few studies have reported on differences in prevalence of iNPH between men and women [11–13]. In accordance with the present study, Jaraj et al. [11] and Tanaka et al. [12] did not find any statistically significant difference in the prevalence between the sexes. A recent study from Spain reported a predominance of men with iNPH, with a rate ratio of approximately 1.5:1, among those who were referred to a specialist for suspected iNPH [13]. This, however, does not necessary reflect the sex distribution of iNPH in the general population.

### Methodological considerations and diagnostic criteria

The varying results between different prevalence studies are probably due to heterogeneity of study design and study populations which have been discussed in a thorough review [17]. Most previous studies were based on retrospective data from cohorts initially investigated for other diseases than iNPH. The disadvantages include lack of tailored questions and objective tests for assessing iNPH symptoms. In addition, retrospective analyses of neuroimaging findings might be challenging, when these were conducted for a disorder not initially considered, and perhaps with outdated techniques.

Furthermore, previous studies have used different diagnostic criteria for iNPH. There are currently two sets of guidelines for diagnosis, the American-European and Japanese (2^nd^ edition) [9, 26]. These differ to such an extent that they give rise to different prevalence figures, for example in our material where the prevalence was more than twice as high with the American-European guidelines than with the Japanese.

In a recent publication, we compared diagnoses according to the two guidelines and a senior consultant in neurology, respectively. A higher agreement was found between the Japanese guidelines and the neurologist (kappa = 0.69) than between the American-European guidelines and the neurologist (kappa = 0.44) [27]. We concluded that one widely recognized, diagnostic system is needed in order to compare different studies. In this study, we report the prevalence figures according both guidelines, since the American-European are the most commonly used outside Japan.

### Strengths and limitations

The strengths of this study include the prospective screening of the general population with a questionnaire specifically designed for iNPH, use of international diagnostic guidelines and standardized testing with an iNPH specific symptom scale. Data from an unselected general population are more likely to reflect the true prevalence and age and sex distribution of the disorder and are more generalizable to other populations. On the other hand, a limitation with questionnaire screening is the lack of information about the non-responders. It might be that individuals with symptoms are more prone to participate, or on the contrary, that the oldest and those with the largest impairment are more likely to decline further participation. The latter was supported by the non-responders being significantly older than the responders. The female predominance among both the non-responders and the responders probably just mirrors the sex distribution in these age groups in the population of Jämtland [18]. Thus, the prevalence presented here, although higher than in previous reports, might still be an underestimation due to a dropout of those most afflicted by the condition.

In this study, we have not performed any opening pressure measurements to ascertain that the lumbar opening pressure was below 25 cm H_2_0, which is currently required for a diagnosis of probable iNPH according to the American-European guidelines. Although this may be considered as a limitation, we believe this criterion is a drawback for large epidemiological studies. In a clinical setting, however, measurements on CSF opening pressure as well as obtaining biomarkers from cerebrospinal fluid is of importance, especially for differential diagnostics.

### Clinical applications

The finding of a prevalence of iNPH of 3.7% in this study contrasts to the incidence of shunt-surgery for iNPH of 2.2 /100, 000/year in Sweden during 2004–2011 [28]. In Norway the incidence of surgery was even lower; 1.09/100, 000 during 2002–2006 [29]. This discrepancy between the prevalence of iNPH and the proportion who obtains treatment suggest that the condition is highly under-diagnosed and under-treated.

Treatment with shunt surgery is effective, especially in an early stage before the patient become wheelchair bound and demented [6], and therefore iNPH patients should be better recognized by the health care system. A simple screening tool for iNPH symptoms could be useful for primary care and patients with symptoms should be further investigated and referred for neuroimaging. Extra attention should be paid to elderly with falling accidents. Since radiological changes of iNPH might be misinterpreted, a radiological screening tool has been developed by our research group to enable structured and objective assessments of images [21].

## Conclusions

In this prospective, population-based study the prevalence of probable iNPH was 3.7% among individuals 65 years and older, and more common in the higher age group, 80 years and above. INPH needs to be increasingly recognized since it is a fairly common condition and an important cause of gait impairment and dementia among the elderly that can, in most cases, be effectively treated by a shunt-operation.

## Supporting information

S1 Questionnaire (PDF)
