# Supplementary material for: Prevalence of idiopathic normal pressure hydrocephalus: A prospective, population-based study
Source: PLoS One. 2019 May 29;14(5):e0217705. doi: 10.1371/journal.pone.0217705 (PMC6541279; doi:10.1371/journal.pone.0217705)
Supplement: S1 Questionnaire — (PDF) [file pone.0217705.s001.pdf]

Förekomst av NPH i Jämtland

Följande frågor är utformade för att bedöma om du kan lida av NPH.
Att ha symptom behöver dock inte innebära att man har sjukdomen NPH, utan diagnosen måste ställas av en läkare.

Var god kryssa i ditt svar på samtliga frågor.

1. Mina fötter känns som klistrade vid underlaget när jag går

- ☐ Ja
- ☐ Nej

2. Jag har svårt att hålla balansen när jag går eller när jag vänder mig om

- ☐ Ja
- ☐ Nej

3. Jag har fallit flera gånger utan att jag förlorat medvetandet

- ☐ Ja
- ☐ Nej

4. Jag har svårt att upprätthålla uppmärksamheten en längre tid

- ☐ Ja
- ☐ Nej

5. Jag har svårt att komma ihåg saker

- ☐ Ja
- ☐ Nej

6. Jag har upplevt plötsligt påkommande urinträngningar och måste fort hitta en toalett/kissat på mig

- ☐ Ja
- ☐ Nej

7. Jag går allt långsammare

- ☐ Ja
- ☐ Nej

8. Lider du av någon annan sjukdom som du vet påverkar din gång eller balansförmåga t.ex. förlamning efter en tidigare stroke eller uttalad förslitning av höfter och/eller knän?

- ☐ Ja
- ☐ Nej

Om du kryssat ja på fråga 8 ange vilken sjukdom som avses:

____

Jag är

- ☐ Kvinna
- ☐ Man

Ålder:..

Eventuell kommentar:

____

____

Detta enkätsvar postas tillsammans med svarsformuläret i medsänt, frankerat svarskuvert.

Tack för din medverkan!

Katarina Laurell, lektor, överläkare i neurologi, Karin Kockum, ST-läkare i radiologi, Michelle Rosell, medicine student
